# High-Frequency Heating Extraction Method for Sensitive Drug Analysis in Human Nails

**DOI:** 10.3390/molecules23123231

**Published:** 2018-12-07

**Authors:** Fumiki Takahashi, Masaru Kobayashi, Atsushi Kobayashi, Kanya Kobayashi, Hideki Asamura

**Affiliations:** 1 Department of Chemistry, Faculty of Science, Shinshu University, 3-1-1 Asahi, Nagano 390-8621, Japan; 2 Research Institute of Scientific Criminal Investigation, Nagano Prefectural Police Headquarters, 3916 Nishijo, Matsushiro, Nagano 381-1232, Japan; fumi.fumi.fufumi.0001@gmail.com (M.K.); fumi.fumi.fufumi.0002@gmail.com (A.K.); 3 Department of Legal Medicine, Shinshu University School of Medicine, 3-1-1 Asahi, Matsumoto, Nagano 390-8621, Japan; asamura@shinshu-u.ac.jp

**Keywords:** nail, curie temperature, high-frequency heating, liquid chromatography–tandem mass spectrometry, caffeine, amlodipine

## Abstract

*Background:* A simple, sensitive, and rapid extraction method based on high-frequency (H-F) heating was developed for drug analysis in human nails. *Methods:* A human nail was placed in a glass tube with an extraction solvent (methanol and 0.1% formic acid; 7:3, *v*/*v*), and a ferromagnetic alloy (pyrofoil) was wrapped in a spiral around the glass tube. Then, the glass tube was placed in a Curie point pyrolyzer, and a H-F alternating voltage (600 kHz) was applied. The sample and extraction solvent were heated at the Curie temperature for 3 min. Different Curie temperatures were applied by changing the pyrofoil (160 °C, 170 °C, 220 °C, and 255 °C). *Results:* The caffeine in the nail was effectively and rapidly extracted into the extraction solvent with the pyrofoil at 220 °C. The peak area obtained for the caffeine using liquid chromatography mass spectrometry (LC-MS/MS) was five times that of what was obtained after conventional ultrasonic irradiation extraction. Because the extraction uses high-pressure and high-temperature conditions in a test tube, the drugs that were strongly incorporated in nails could be extracted into the solvent. The amount of caffeine extracted was independent of the size of the pieces in the sample. *Conclusions:* Therefore, the sensitive determination of target drugs in nails is possible with rapid (20 min, including H-F extraction for 3 min) and simple sample preparation. The developed method was applied to a nail from a patient with hypertension.

## 1. Introduction

Blood, urine, and gastric contents are commonly used for drug analysis in forensic and clinical toxicology, and allow for a relatively easy estimation of toxicological risk [1,2,3,4]. However, when these samples are not available, such as after the putrefaction and decomposition of the body, hair and nails can be used as alternative samples for forensic and biomedical analysis [5,6]. These alternative specimens provide a long surveillance window for exposure, because drugs incorporated into the hair and nails have a high stability at room temperature [7,8]. Therefore, many applications of hair samples for drug detection have been reported for drugs of abuse, such as methamphetamine [9,10,11], opiates [12], and cannabis [13], as well as therapeutic drugs, such as benzodiazepines and barbiturates [7,14]. However, on the other hand, there has been little investigation of nail samples for drug analysis. Because nails are harder than hair, the effective extraction of drugs from nails is very difficult. Some reports on drug analysis in nails have used alkaline or enzymatic hydrolysis extraction, but these methods have long extraction times and result in sample destruction [15,16]. Moreover, an effective drug extraction method is required in order to determine the concentrations of drugs in nails at trace levels [6,17,18].

High-frequency (H-F) induction heating is a pyrolysis technique that uses a ferromagnetic alloy and a H-F induction coil [19,20,21]. The ferromagnetic alloy is placed in an electromagnetic field with a H-F generated using a pyrolyzer, and the alloy is heated to its Curie temperature. The Curie temperature can be reached in as little as a few hundredths of a second. Hence, the combination of H-F heating pyrolysis with GC-MS has been accepted as a powerful tool for the analysis of rubber, plastic, and resin [20,22]. Recently, Kurihara et al. reported the analysis of additives in polymers using H-F heating extraction [23]. Polymers were placed in a glass tube with glass wool, and the tube was wrapped with a ferromagnetic alloy. Subsequently, a H-F alternating voltage was applied to the glass tube, and the additives in the polymer were rapidly extracted onto the glass wool. The sensitive detection of the extracted additives was performed by gas chromatography (GC)-MS. Thermal extraction with H-F heating was applied to the fatty acids [24], antioxidants [25], and polymer resins [26,27].

In our previous work, H-F extraction was applied to drug analysis in nails [28]. Human nails were placed in a glass tube with a solvent, and were heated as described above. A highly effective solvent extraction from the nails was achieved, and this was combined with liquid chromatography mass spectrometry (LC-MS/MS). This approach is simple, rapid, and does not need a sample decomposition such as alkalization. The proposed method has high sensitivity compared with the conventional non-destructive method of ultrasonic irradiation extraction, and the caffeine, amlodipine, probucol, cilostazol, and minoxidil in nails were extracted to the solvent effectively [28]. However, the extraction processes have not been examined quantitatively, including the morphological approach. Because information regarding the morphology of the nail undergoing the H-F heating process is one of the essential solid–liquid solvent extraction process, this knowledge provides the optimization for drug analysis in nails. In addition, the incorporation of drugs to nails under metabolic processes, especially the effect of sample site (finger- and toe-nail), has not been characterized.

In this study, the H-F heating extraction method was optimized using caffeine as a representative drug. Caffeine is an ingredient in stimulant preparations and is often used as an adulterant in drugs of abuse [6]. The effect of the H-F heating process was evaluated using morphological and metabolomic information, the proposed extraction technique had suitability for drug analysis in human nails, because the incorporated property of the drugs is highly bound to the nail. The optimized method was applied to the sensitive determination and characterization of amlodipine in toenails and fingernails from a patient with hypertension.

## 2. Results and Discussion

### 2.1. Features of H-F Heating Extraction

**Figure 1:**
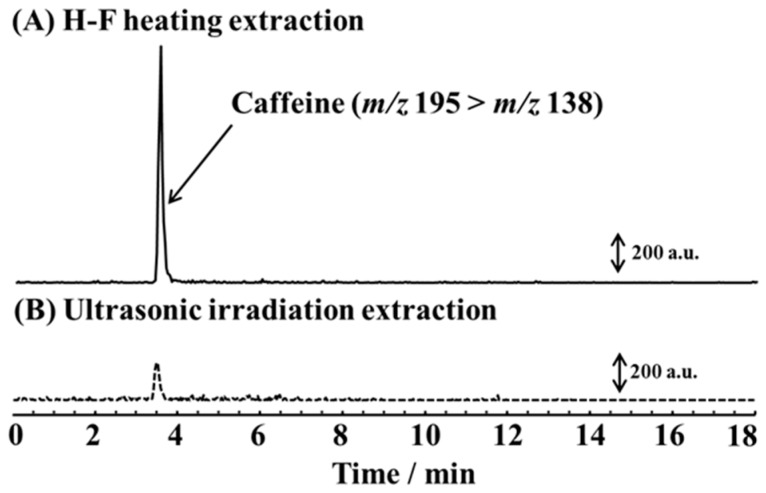
Selected reaction monitoring (SRM) chromatogram for caffeine in nails with (**A**) high-frequency (H-F) heating extraction (3 min; 220 °C) and (**B**) ultrasonic irradiation extraction (30 min). The sample mass was 10 mg and the extraction solvent volume was 200 μL.

In the initial experiments, we applied the H-F heating extraction method to caffeine. A selected ion monitoring (SIM) chromatogram (Figure 1) was obtained of a sample extracted into 200 μL of solvent from 10 mg of nails.

The H-F heating extraction time was fixed at 3 min, and the Curie point temperature was 220 °C (Figure 1A). For the validation of the proposed method, a conventional ultrasonic extraction with indirect irradiation was carried out for 30 min (Figure 1B). The peak at 3.5 min in the SIM chromatogram was assigned to caffeine. The H-F heating method showed a much better sensitivity for caffeine than the ultrasonic extraction method, and the peak area of the caffeine in the SIM chromatogram was more than five times that obtained using the conventional extraction. The incorporated property of the drugs is highly bound to nails, and the effective extraction of drugs was suggested from nails.

To evaluate the H-F heating process, the surface profiles of the nails under extraction were evaluated using SEM.

**Figure 2:**
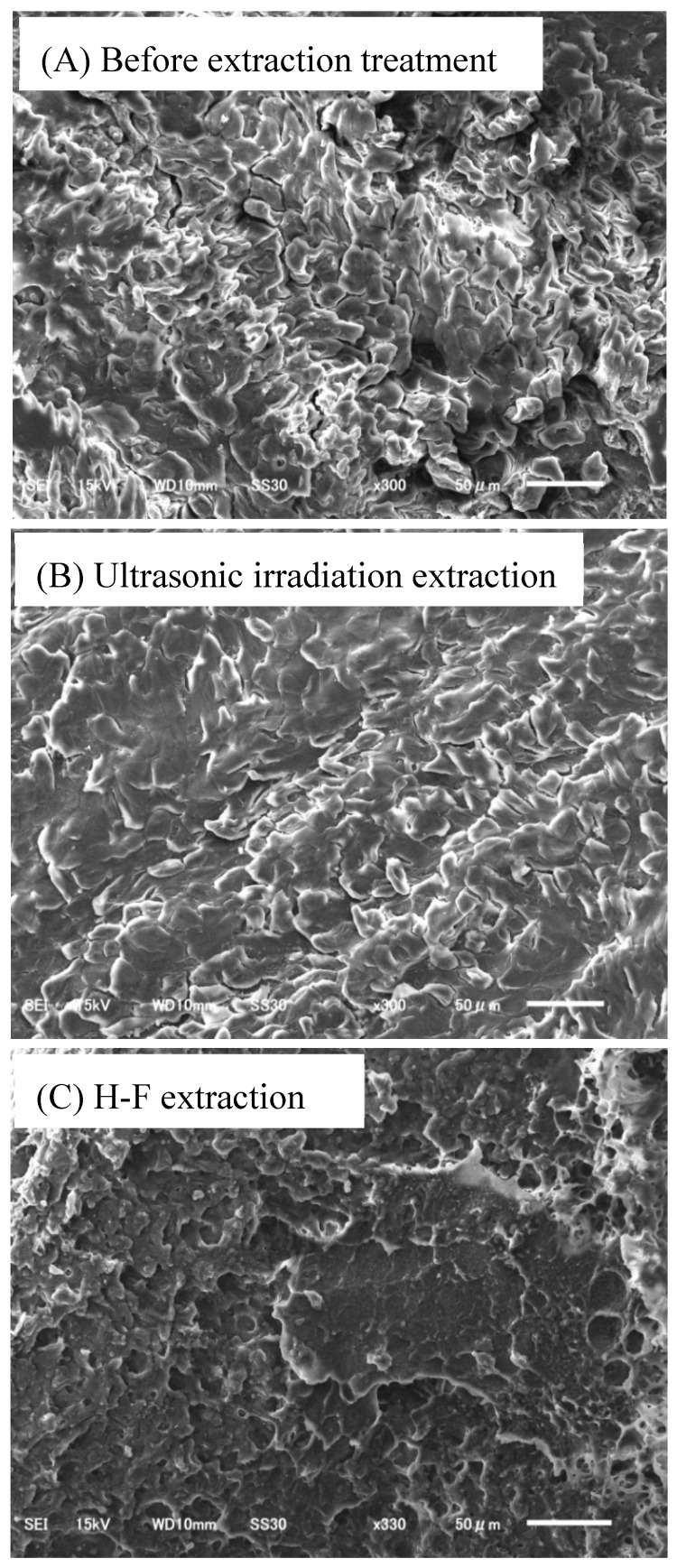
SEM images of the nail surface (**A**) before the extraction treatment, (**B**) after ultrasonic irradiation extraction (30 min), and (**C**) after H-F heating extraction (3 min).

The nail before extraction was constructed of fine plates that were arranged in layers on the nail surface (Figure 2A). This structure was maintained even after ultrasonic irradiation extraction for 30 min (Figure 2B). This illustrates that ultrasonic irradiation has almost no effect on the nail surface structure, which could explain why it is difficult to effectively extract drugs from nails using the conventional extraction method. However, after H-F heating extraction (220 °C, 3 min), holes appeared on the nail surface (Figure 2C). This shows that the nail structure was affected by the high-temperature and high-pressure conditions generated by the H-F heating in the glass tube. This suggests that the effective extraction of caffeine using H-F heating is related to the increased solid–liquid surface area between the nails and the extraction solvent. However, the fundamental shape of the nail was retained, and non-destructive extraction with a high sensitivity could be achieved using the H-F heating method.

### 2.2. Optimization of H-F Heating Extraction for Nail Analysis

To optimize the H-F heating extraction, the dependence of the peak area of the caffeine on the extraction conditions was evaluated. The nail mass was 10 mg and the extraction solvent volume was 200 μL. 

**Figure 3:**
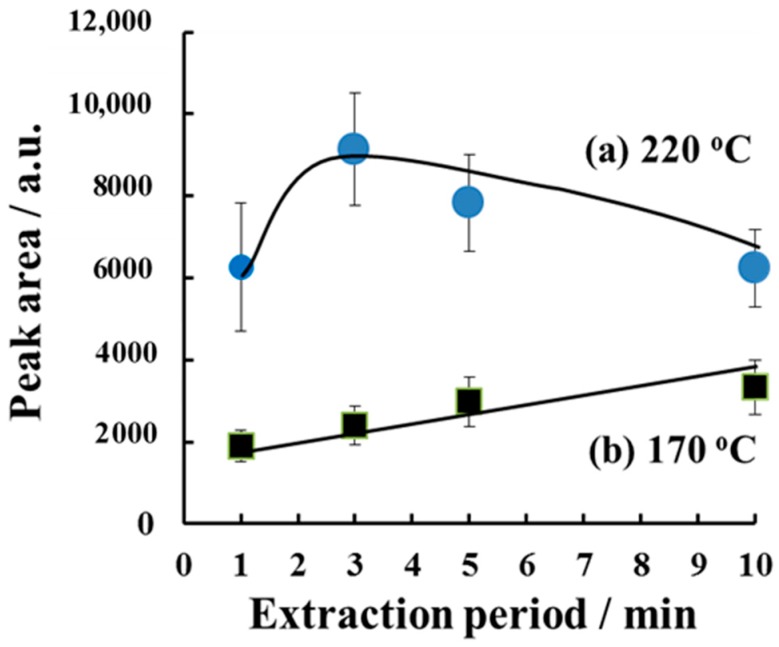
Dependence of the caffeine peak area on the extraction time for extraction at 220 °C ((a) closed circles) and 170 °C ((b) closed squares), respectively. The sample mass was 10 mg and the extraction solvent volume was 200 μL. Error bars represent the standard deviation (*n* = 3).

When we plotted the caffeine peak area as a function of the extraction time for the H-F heating method (Figure 3), we found the caffeine peak area increased with the increase in the extraction time at 170 °C, but the signal was relatively low. By contrast, at 220 °C, the caffeine contained in the nails was rapidly extracted into the solvent within a short time. At this temperature, the largest peak area was obtained with a 3-min extraction time. The amount of caffeine extracted decreased slightly as the extraction time was increased to over 5 min. If the extraction period is too short, the drugs in the nail will not be completely extracted into the solvent. The optimum time for the H-F heating extraction was 3 min.

It is well known that nails contain keratinized proteins in fine plates [6,29], but the chemical and physical properties of nails have not been completely characterized. Fujii et al. fabricated novel keratin fibers and films from actual human hair and nails, and evaluated their chemical stability under thermal treatment [16,30,31]. The chemical structure of the nail was denatured at temperatures of over 170 °C [30]. In the present study, morphological changes occurred and small holes were generated at the nail surface, which enhanced the extraction of the drug into the solvent at 220 °C under H-F heating. On the other hand, temperatures that were too high (255 °C) decreased the caffeine signal (Figure S1), because the thermal decomposition of cthe affeine was reported at 237 °C using differential scanning calorimetric (DSC) analysis [32]. In addition, the fundamental nail structure was destroyed and the solvent became cloudy as the nail dissolved at 255 °C. Caffeine may be incorporated into small particles of the nail rather than extracted into the solvent, and the caffeine signal will decrease. 

**Figure 4:**
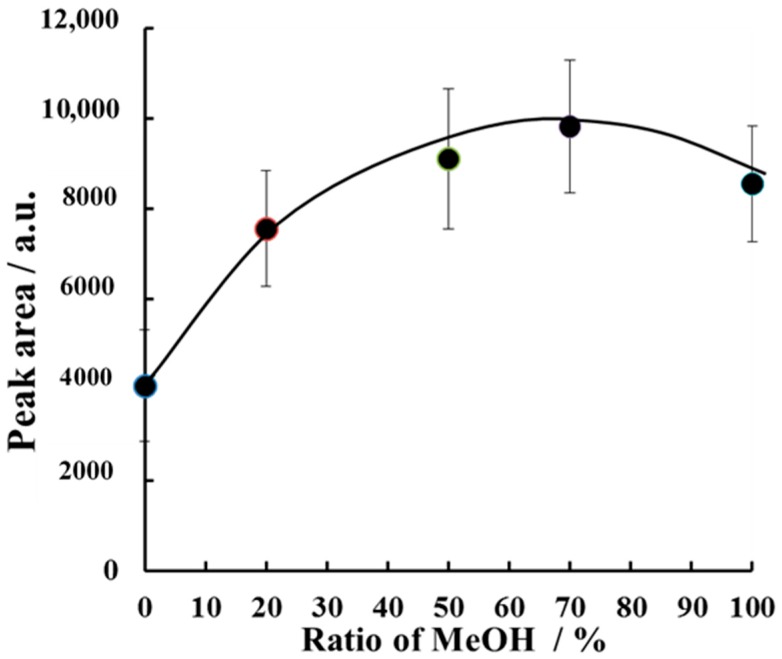
Dependence of the caffeine peak area on the ratio of MeOH in the extraction solvent for H-F heating extraction. The sample mass was 10 mg and the H-F heating extraction was carried out for 3 min at 220 °C. Error bars represent the standard deviation (*n* = 3).

Next, the dependence of the caffeine peak area on the ratio of methanol (MeOH) in the extraction solvent was evaluated (Figure 4).

When we used only 0.1% formic acid with no MeOH, a relatively small amount of caffeine was extracted into the solvent. The caffeine peak area increased as the proportion of MeOH increased to 70%. The highest amount of caffeine was extracted with 70% MeOH. When 100% MeOH was used as the extraction solvent, the extraction procedure was relatively difficult, because of the risk of rupture of the glass tube under H-F heating. Therefore, methanol-0.1% formic acid (7:3, *v*/*v*) was selected as the extraction solvent.

**Figure 5:**
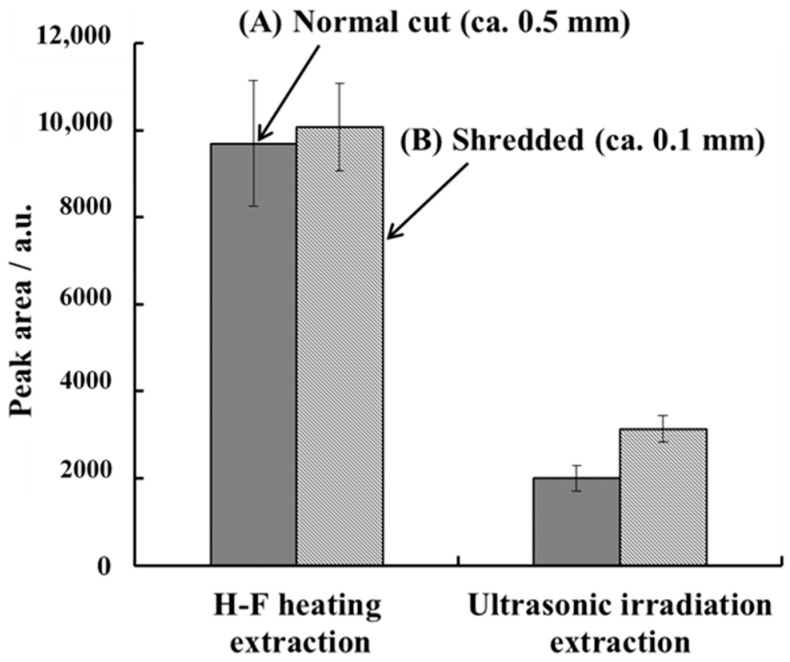
Effect of the size of pieces in the sample (((A) fill pattern = dots) cut to 0.5 mm, ((B) fill pattern = diagonal lines) shredded to 0.1 mm) on the caffeine peak area after H-F heating extraction and ultrasonic irradiation extraction. The sample mass was 10 mg and the extraction solution volume were 200 μL. The other parameters were the same as in Figure 4. Error bars represent the standard deviation (*n* = 3).

The dependence of the caffeine peak area on the size of the pieces in the nail sample was investigated by cutting (ca. 0.5 mm) and shredding (ca. 0.1 mm) the samples (Figure 5).

With the ultrasonic irradiation extraction method, the amount of caffeine extracted from the shredded sample was 50% more than that obtained from the cut sample. The surface area of the sample increased after shredding, which could improve the efficiency of the solid–liquid extraction of caffeine into the solvent. A sample pretreatment to increase the surface area is required for the sensitive determination of caffeine using the ultrasonic extraction method [33]. By contrast, in the H-F heating extraction method, no significant changes were observed between the cut and shredded samples for the amount of caffeine extracted. The caffeine incorporated in the nail was effectively extracted into the solvent using H-F heating under high-temperature and high-pressure conditions for both types of samples. Therefore, sample shredding may not be essential for H-F heating extraction. These results show that the H-F heating extraction method can be performed using a simple sample preparation method without any decrease in the sensitivity. 

### 2.3. Application of H-F Heating Extraction to Nails from a Hypertension Patient 

**Figure 6:**
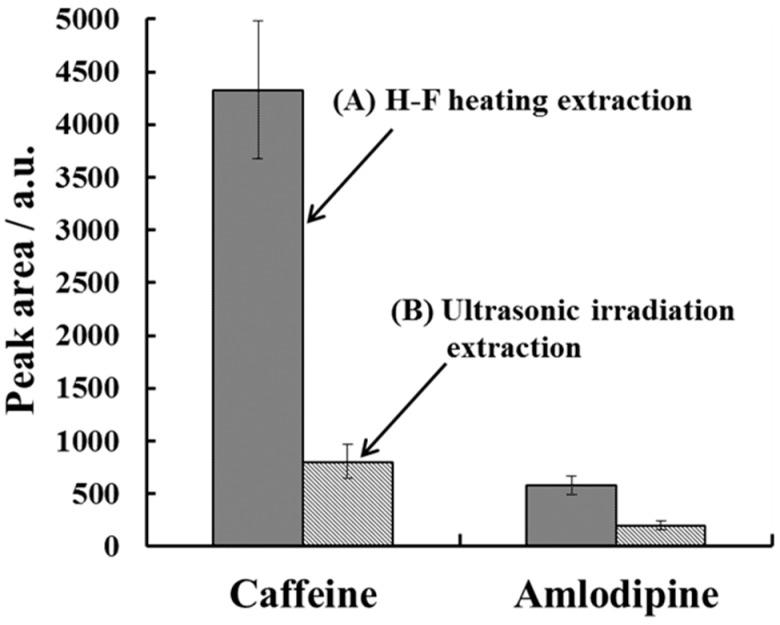
The extraction profiles of caffeine and amlodipine for nails from a hypertension patient. (A) H-F heating extraction (3 min; 220 °C) and (B) ultrasonic irradiation extraction (30 min). The other parameters were the same as in Figure 5. Error bars represent the standard deviation (*n* = 3).

The H-F heating extraction was applied for the detection of amlodipine in nails from a hypertension patient. Caffeine and amlodipine were extracted using H-F heating and ultrasonic irradiation (Figure 6). 

In the H-F heating extraction results, the peak areas for both caffeine and amlodipine were higher than those obtained with the ultrasonic irradiation method. This result shows that multiple drugs can be effectively extracted into the solvent using H-F heating. 

**Figure 7:**
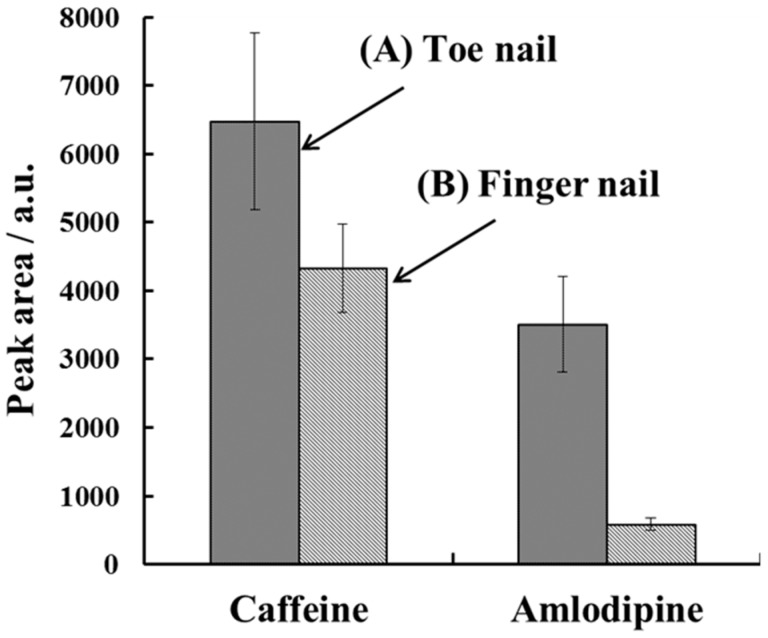
Comparison of the concentrations of caffeine and amlodipine in (A) toenails and (B) fingernails from a hypertension patient. The H-F heating extraction conditions were the same as in Figure 6A. Error bars represent the standard deviation (*n* = 3).

The peak areas of the caffeine and amlodipine from the toenails and fingernails were determined after H-F heating extraction (Figure 7). 

Both the caffeine and amlodipine were more concentrated in the toenails than in the fingernails. Drugs are deposited in the nails from blood or sweat during the germinal matrix generation of nails [29,33]. The growth rates of the fingernails and toenails are approximately 3–5 mm/month and approximately 1 mm/month, respectively [6,29]. Consequently, for samples of the same mass, the toenails will have been exposed to any drugs in the system for longer than fingernails. This correlates with the larger peak signals measured for the toenail samples. However, the concentration ratio for caffeine between the toenails and fingernails was higher than that for amlodipine. It is likely that the external contamination of the nails with caffeine powder increased the caffeine signal. These results show that the toenail analysis may be advantageous over the fingernail analysis for the detection of drugs in cases of continuous drug use. However, the incorporation mechanisms of the drugs in the nails are unclear, and are being examined in our laboratory currently. 

The total time required for the extraction of one sample was less than 20 min, with 3 min for the H-F heating extraction. Compared with the conventional ultrasonic irradiation, this time is a shorter and should improve the analytical reliability without the need for sample shredding to increase the surface area. The H-F heating extraction is also advantageous compared with the conventional method, because all of the extraction procedure is carried out in a glass tube without the need for an enzyme or strong reagent. Therefore, the extraction of drugs from nail samples using H-F heating extraction is simpler and should have a lower risk of sample contamination. The correct determination of drugs in nails is practically difficult, because the extraction efficiency influences the quantitative results. However, the proposed method provides an improvement on the extraction efficiency from nails, and the results are useful when the sample decomposition cannot be available. This method will expand the potential applications of forensic and clinical toxicology analysis.

## 3. Materials and Methods 

### 3.1. Chemicals

Analytical grade caffeine and amlodipine were purchased from Wako Pure Chemical Industries Ltd. (Osaka, Japan), and were used as received. HPLC grade 0.1% (*v*/*v*) formic acid and 0.1% (*v*/*v*) formic acid-acetonitrile were purchased from Kanto Chemical Ltd. (Tokyo, Japan). All of the other reagents were of the highest grade available, and were purchased from Tokyo Chemical Industry Co. Ltd. (Tokyo, Japan). Stock solutions (200 μg/mL) of caffeine and amlodipine were prepared with methanol, and stored at −20 °C when not in use. Working standard solutions were prepared by the precise dilution of the stock solutions using methanol just before they were required. Water was purified using a Milli-Q Integral purification system (Millipore, Billerica, MA, USA).

### 3.2. Apparatus

The H-F heating extraction was carried out using a ferromagnetic alloy and a Curie point pyrolyzer (JHP-3S, 600 kHz, Japan Analytical Industry Co. Ltd., Tokyo, Japan). Different ferromagnetic alloys (160 °C, 170 °C, 220 °C, and 250 °C) were purchased from Japan Analytical Industry (Tokyo, Japan). Glass tubes (70 mm × 5 mm i.d.) with one sealed end were purchased from Shimadzu GLC (Tokyo, Japan). A BRASONIC ultrasonic cleaner (80 W; 47 kHz; BRANSON, Danbury, CT, USA) was used for the conventional ultrasonic irradiation extraction [26].

The LC-MS/MS analyses were performed using a Prominence UFLC liquid chromatograph (Shimadzu Co., Kyoto, Japan) combined with a LXQ mass spectrometer (Thermo Fisher Scientific, MA, Waltham, MA, USA). An Inersil^®^ ODS-4 column (75 mm × 2.1 mm i.d.; 3 μm particle size; GL Science Inc., Tokyo, Japan) was used for separation, and kept at 40 °C during the analysis. The mobile phase was a mixture of 0.1% (*v*/*v*) formic acid (solvent A) and 0.1% (*v*/*v*) formic acid-methanol (solvent B). Both components of the mobile phase were filtered and degassed before use. The elution used a linear gradient of solvent B from 20% to 80% (20 min), with a constant mobile phase flow rate of 0.2 mL min^−1^. The mass spectra were collected in MS/MS mode. The precursor ion *m*/*z* 195 was selected for caffeine and *m*/*z* 409 for amlodipine. The product ions were analyzed in the selected reaction monitoring (SRM) mode. The ions at *m*/*z* 138 and *m*/*z* 238 were selected for the detection of caffeine and amlodipine, respectively.

Scanning electron microscope (SEM) images were obtained by a JSM-6610LV electron microscope (JEOL, Tokyo, Japan), and used to observe the surface profiles of the nails.

### 3.3. Sample Preparation 

**Figure 8:**
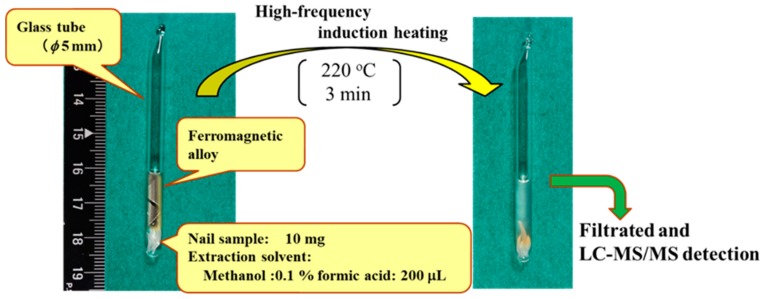
Schematic diagram of the high-frequency (H-F) heating extraction method.

Nails were collected from two adult volunteers with their agreement under informed consent. One volunteer (thirties; male) constantly consumed caffeine during daily life. The other volunteer (forties; male) consumed caffeine and took amlodipine as an antihypertensive (10 mg/day). Both their finger- and toe-nail samples were collected using commercial nail clippers during daily hygiene routines. The collected nail samples were cut into 1-mm segments, and mixed until homogenous. Then, the nail samples were washed by ultrasonication with 1% sodium dodecyl sulfate solution for 1 min, with water five times, and then with methanol. After washing, the nails were air dried at room temperature. Figure 8 shows a schematic diagram of the H-F heating extraction method.

The nails (10 mg) were placed in glass tube with 200 μL of the extraction solvent and the glass tube was sealed over a flame. Next, a ferromagnetic alloy (pyrofoil; 9 mm × 20 mm, Shimadzu GLC Co. Ltd., Tokyo, Japan) was wrapped around the glass tube in a spiral, and the glass tube was placed in the pyrolyzer. A H-F voltage (600 kHz) was applied to the pyrofoil for a few minutes, and any drugs contained in the nails were extracted into the solvent. The extraction temperature was the Curie temperature of the pyrofoil. After extraction, the glass tube was cooled to room temperature. Then, the glass tube was cut through the middle, and the extraction solvent was collected using a microsyringe, and was filtered through a Mini-Uniprep syringeless filter device (pore size 0.45 μm, GE Healthcare UK Ltd., Buckinghamshire, UK). Five microliters of the extract were injected into the LC-MS/MS system.

Part of this work was carried under ethics certification (No. 25M3) from the Japanese Association of Forensic Science and Technology.

## 4. Conclusions

In this study, a simple and efficient extraction method for the analysis of drugs in nails was developed using H-F heating combined with LC-MS. A significant peak for caffeine was obtained by the proposed method using MeOH:0.1% formic acid (7:3, *v*/*v*) as the extraction solvent. Because of the high-pressure and high-temperature conditions in the glass tube under H-F heating, the drugs that are strongly incorporated in the nails will be effectively extracted into the solvent within 3 min. Under the optimized extraction conditions, the extraction of the drugs is not dependent on the sample preparation process (e.g., cutting or shredding). Therefore, H-F heating will be useful for simplifying extraction in forensic and clinical analysis, and this will reduce the overall extraction time and minimize the destruction of the sample. This method was successfully applied to the detection of drugs in nails from a hypertension patient.

## Supplementary Materials

The following are available online. Figure S1: Dependence of the caffeine peak area on the extraction temperature for extraction of caffeine from nails.
