# Supplementary material for: High-Frequency Heating Extraction Method for Sensitive Drug Analysis in Human Nails
Source: Molecules. 2018 Dec 7;23(12):3231. doi: 10.3390/molecules23123231 (PMC6320838; doi:10.3390/molecules23123231)
Supplement: Supplementary file 1 [file molecules-23-03231-s001.pdf]

Electronic Supplementary Material

High-frequency Heating Extraction Method for Sensitive Drug Analysis in Human nails

Fumiki Takahashi [1,*], Masaru Kobayashi [2], Atsushi Kobayashi [2], Kanya Kobayashi [3,*] and Hideki Asamura [3]

1 Department of Chemistry, Faculty of Science, Shinshu University, 3-1-1 Asahi, Nagano 390-8621, Japan
2 Research Institute of Scientific Criminal Investigation, Nagano Prefectural Police Headquarters, 3916 Nishijo, Matsushiro, Nagano 381-1232, Japan; takahashi@shinshu-u.ac.jp
3 Department of Legal Medicine, Shinshu University School of Medicine, 3-1-1 Asahi, Matsumoto, Nagano 390-8621, Japan; kanya_k@shinshu-u.ac.jp
* Correspondence: takahashi@shinshu-u.ac.jp (F.T.); Tel.: +81-263-37-2474
kanya_k@shinshu-u.ac.jp (K.K.); Tel.: +81-263-37-2474

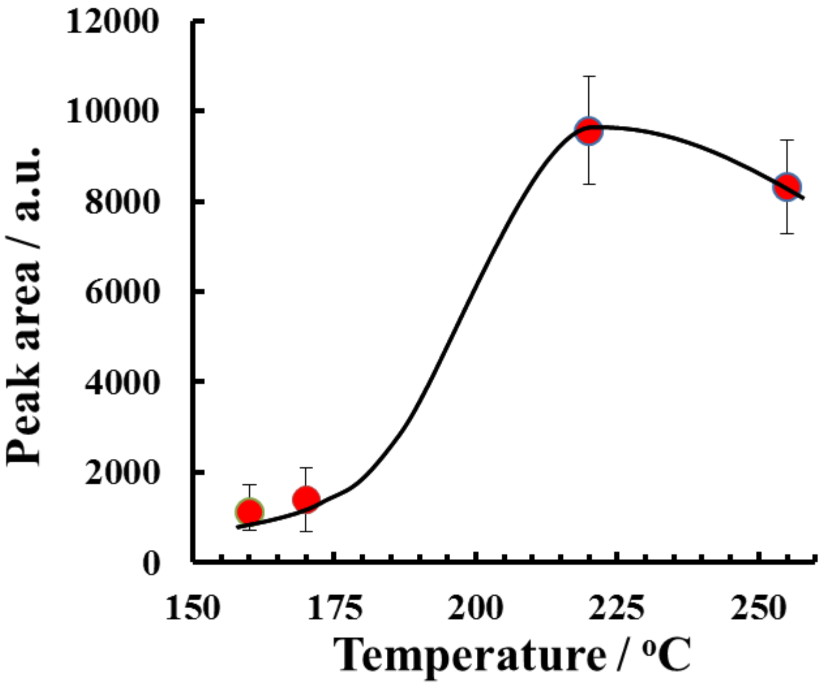

Figure S1. Dependence of the caffeine peak area on the extraction temperature for extraction of caffeine from nails. The sample mass was 10 mg and the H-F heating extraction was carried out at 220 °C. Error bars represent the standard deviation (n = 3).

Figure S1. F. Takahashi
